# A nationwide web-based survey of oncologic surgeons to clarify the current status of preoperative assessment for elderly cancer surgery patients in Japan

**DOI:** 10.1038/s41598-021-02319-7

**Published:** 2021-11-23

**Authors:** Daisuke Inoue, Makoto Yamamoto, Hisatomi Arima, Kazuo Tamura, Yoshio Yoshida

**Affiliations:** 1 Department of Obstetrics and Gynecology, University of Fukui, 23-3 Matsuoka-Shimoaizuki, Eiheiji-cho, Yoshida-gun, Fukui, 910-1193 Japan; 2 Department of Preventive Medicine and Public Health, Fukuoka University, 7-45-1 Nanakuma, Jhonan-ku, Fukuoka, Fukuoka Japan; 3 Emeritus Professor, Faculty of Medicine, Fukuoka University, 7-45-1 Nanakuma, Jhonan-ku, Fukuoka, Fukuoka Japan

**Keywords:** Cancer, Medical research, Oncology

## Abstract

Elderly cancer patients requiring surgical treatment are increasing, and the deterioration of quality of life and shortening of healthy life expectancy due to postoperative complications represent major problems. This study investigated the current status of medical treatment, including perioperative evaluations, for elderly cancer patients requiring surgical treatment at cancer treatment facilities nationwide. A total of 436 cancer care facilities around Japan were invited to participate in this web-based survey regarding management of cancer patients ≥ 65 years old who had undergone surgical treatment in 2018. A total of 919 department heads from 245 facilities agreed to participate. Although most respondents answered that performance status, preoperative examinations, and comorbidities were important when deciding on a treatment plan, age, Geriatric Assessment (GA), and guidelines were "not important" for > 10% of all respondents. GA was familiar to 195 department heads (21%), and awareness of GA was significantly lower among respondents from medical education institutions than the other types of hospitals (18.5% vs 26.3%; P = 0.006). This large survey revealed that the use of GA is not widespread, and its awareness in medical education institutions remains low. We believe that accumulating evidence of geriatric oncology surgery is an urgent issue in Japan.

## Introduction

With the current hyper-aging society present in Japan, mean age at onset of cancer is over 60 years, and 85% of cancer deaths occur in people ≥ 65 years old^1,2^. Cancer is already a chronic disease of the elderly, and its treatment and care require the establishment of systems applicable not only to the medical community, but also to society as a whole. Declines in physiological functions, complications, comorbidities, declines in cognitive functions, and socioeconomic limitations such as institutionalization and living alone need to be taken into account when considering treatments in the elderly^3^. These characteristics are thought to impact therapeutic efficacy and adverse events, and personalized medicine after pre-treatment evaluations is thus desirable. In particular, the number of elderly cancer patients requiring surgical treatment is increasing, and deterioration of quality of life (QOL) and shortening of healthy life expectancy due to postoperative complications represent major problems, and the establishment of treatment guidelines for elderly cancer patients is urgently needed.

The most important issue in the treatment of elderly cancer patients is to perform pre-treatment evaluations, but no consensus has yet been reached regarding the optimal methods for such evaluations and little evidence is currently available^4–6^. Comprehensive Geriatric Assessment (CGA) is a method for comprehensively assessing functional impairment in the elderly^7^. CGA is a concept that combines assessment and intervention, and in oncology is referred to as Geriatric Assessment (GA), in the sense that only a comprehensive assessment is performed to determine a treatment plan^8^. GA is capable of assessing the elderly individual objectively and comprehensively, but is not widely used in daily practice in Japan, especially in cancer treatment. Previously, we have shown that Japanese gynecologic oncology doctors have lower awareness of CGA^9^. However, whether GA has been widely applied in surgical departments other than gynecologic oncology is unknown. For this background, we surveyed the actual status and problems of perioperative evaluation for elderly cancer patients in Japan, and to collect basic data to develop treatment guidelines for elderly cancer patients. This study was conducted as an activity of the "Research on the infrastructure for development of clinical guidelines for elderly cancer patients" group as part of the Research Project for the Promotion of Cancer Control of the Ministry of Health, Labour and Welfare Sciences.

## Materials and methods

This nationwide survey of cancer care facilities was conducted from May to August 2020. Targets of the survey were all 393 designated cancer hospitals by the Ministry of Health, Labor and Welfare [51 prefectural base hospitals for cancer care, 14 regional base hospitals for cancer care (advanced type), 325 regional cooperation base hospitals for cancer care, 1 base hospital for cancer care in a specific area, and 2 National Cancer Centers], and 43 regional base hospitals for cancer care. The following data were collected at a total of 436 facilities (as of April 1, 2019), including 43 regional cancer hospitals, for departments categorized into 12 types (neurosurgery, respiratory surgery, cardiac surgery, gastrointestinal surgery, hepatobiliary surgery, breast surgery, urology, gynecology, orthopedics, otorhinolaryngology/oral oncology, dermatology/plastic surgery, and others) during the year from the beginning of January to the end of December 2018. The survey investigated the management of patients with cancer ≥ 65 years old who underwent surgical treatment during the same period. The survey was sent by mail and e-mail to hospital directors of the target facilities, requesting cooperation in the questionnaire, and asking the head of each department to respond via the web.

### Survey items

#### Background characteristics of respondents

Data requested included: years of experience as a physician; whether the respondent had obtained a medical specialty; whether the respondent had obtained a Ph.D. degree; the types of institution to which the respondent belonged; the specialty of the respondent, and the percentage of elderly treated at each facility.

#### Do you know and implement the geriatric assessment (GA)?

We asked respondents to answer yes or no to whether they knew about GA and, moreover, whether they were practicing it. GA in this question was defined as a method to comprehensively evaluate physical, mental, and social functions.

#### Items to be evaluated when deciding surgical treatment policy for elderly cancer patients

We asked respondents to respond to the following 11 items using a 3-point scale of "very important," "important," or "not important: (1) age; (2) performance status (PS); (3) judgment of the anesthesiologist; (4) results of general preoperative examinations; (5) comorbidities; (6) social factors such as institutionalization or living alone; (7) complications of dementia; (8) overall evaluation of the elderly; (9) severity of sarcopenia; (10) guidelines; and (11) family wishes.

#### Preoperative evaluations performed and what methods of evaluation were used?

Data on whether the following eight preoperative assessment items were performed and how they were assessed were requested: (1) physical function; (2) comorbidities; (3) medications; (4) nutritional status; (5) cognitive function; (6) depression; (7) social support status; and (8) risk of delirium.

### Statistical analysis

Among the survey items, to examine associations of "presence of GA awareness" with "years of experience as a physician," "whether the respondent had obtained a medical specialty," "whether the respondent had obtained Ph.D.," "the facility to which the respondent belonged," "the specialty of the respondent," and "the annual percentage of elderly treated at each facility," chi-squared tests were used. The level of statistical significance was set at less than 5%. IBM SPSS Statistics 27 (IBM, Armonk, NY, USA) was used for all statistical analyses.

### Ethics

All study protocols were conducted with the approval of The Research Committee of the University of Fukui (approval number 20190123). Since this was a retrospective observational study, the need for written informed consent was waived by the Certified Review Board of the University of Fukui (CRB5180014). Instead of written informed consent, information about this survey was published and participants were given the right to opt out. This survey was conducted in accordance with the Declaration of Helsinki. All methods were performed in accordance with the approved guidelines and regulations.

## Results

We asked each department of the 436 facilities to cooperate in completing the questionnaire, and received responses from 245 facilities (56%), with a total of 941 department heads. Of these, 22 department heads declined to participate in the study and valid responses were obtained from 919 department heads.

Background characteristics of respondents (Table 1).

**Table 1:** Background characteristics of respondents.

	*N* = 919 (%)
**Years of experience as a physician**	
1–15	81 (8.8)
16–20	121 (13.2)
21–25	233 (25.4)
26–	484 (52.6)
Medical specialist	894 (97.3)
Ph.D. degree	778 (84.7)
**Respondent's department**	
Gastrointestinal surgery	122 (13.3)
Dermatology/plastic surgery	120 (13.1)
Otorhinolaryngology/oral oncology	117 (12.7)
Gynecology	97 (10.5)
Respiratory surgery	93 (10.1)
Urology	87 (9.5)
Breast surgery	68 (7.4)
Orthopedics	67 (7.3)
Hepatobiliary surgery	61 (6.6)
Cardiac surgery	20 (2.2)
Neurosurgery	11 (1.2)
Others	56 (6.1)
**Percentage of surgical patients**	Mean (SD)
< 65 years	35.5 (21.7)
65–74 years	31.9 (13.2)
75–84 years	24.6 (13.5)
85 < years	8.1 (9.3)

All respondents answered to these questionnaires. In terms of institutional classifications, 594 (64.6%) department heads belonged to 66 medical education institutions such as university hospitals, 323 (35.2%) belonged to 177 hospitals other than medical education institutions, and one each belonged to a clinic, or government institution or company (including industrial physicians). Gastrointestinal surgery was the most common department, with 122 respondents (13.3%). The average percentages of patients < 65 years old, 65–74 years old, 75–84 years old, and ≥ 85 years old were 36.3%, 32.5%, 25.1%, and 9.0%, respectively. The average proportion of cancer surgery patients ≥ 65 years old was higher in medical education institutions than other hospitals (66.2% vs 61.4%, P = 0.001).

**Figure 1:**
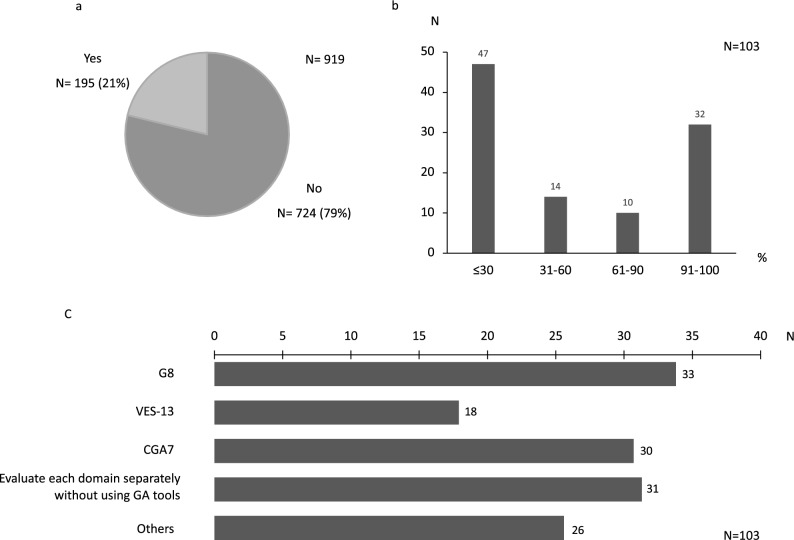
GA recognition and implementation rates, and assessment tools. (**a**) GA recognition rate. (**b**) GA implementation rate. (**c**) GA evaluation tools. (Multiple responses accepted). *GA* geriatric assessment.

GA (Fig. 1, Table 2).

**Table 2:** GA awareness and respondent background.

	Number of GA recognition	%	P-value
**Years of experience as a physician**			
1–15	12/81	14.8	0.060
16–20	21/121	17.4	
21–25	43/233	18.5	
26–	119/484	24.6	
Medical specialist	191/894	21.4	0.518
Ph.D. degree	176/778	22.6	0.015
**Facilities**			
Medical and educational institutions (university hospitals, etc.)	110/594	18.5	0.006
Hospitals (excluding medical and educational institutions)	85/323	26.3	
**Percentage of elderly treated**			
0–25%	8/42	19.0	0.695
26–50%	38/154	24.7	
51–75%	69/328	21.0	
76–100%	80/396	20.3	

*GA* geriatric assessment.

All respondents answered to the questionnaire about whether they know GA. Of the 919 valid responses, 195 department heads (21%) answered they knew about GA. Among those, 103 (11% of the total) answered they were actually performing GA. Furthermore, only 30 respondents indicated that they were implementing GA in all cases.

The chi-squared test was used to examine whether GA awareness was associated with respondent background. Respondents from medical education institutions were significantly less likely to be aware of GA (18.5%) than those from other hospitals (26.3%; P = 0.006). This result can be interpreted as meaning that medical education institutions have a lower awareness of GA. With regard to the relationship between whether a respondent had obtained a Ph.D. degree and GA, the results can be interpreted as indicating that doctors who had obtained a Ph.D. degree were more aware of GA (P = 0.015). In addition, the more years of experience a physician had, the more likely they were to be aware of GA. No significant relationships were apparent between specialty certification, percentage of institutional elderly treated, and awareness of GA. A significant association was seen between GA awareness and department type (P < 0.001), with awareness higher in departments of gastrointestinal surgery and urology, and lower in departments of orthopedics and dermatology/plastic surgery. Regarding the implementation of GA, the 103 respondents who answered that they were implementing GA were 47/278 (14.2%) of medical education institutions and 57/538 (9.6%) of other hospitals (P = 0.033). Medical education institutions were implementing more GA.

Items considered important when deciding on a treatment plan (Table 3).

**Table 3:** Questions and answers on surgical treatment decisions for elderly cancer patients.

	Very important n (%)	Important n (%)	Not important n (%)
Age	170 (18.5)	640 (69.6)	109 (11.9)
Performance status	685 (74.5)	221 (24.0)	13 (1.5)
Anesthesiologist opinion	380 (41.3)	495 (53.9)	44 (4.8)
Preoperative examination	469 (51.0)	437 (47.6)	13 (1.4)
Complications	575 (62.6)	340 (37.0)	4 (0.4)
Social factor	224 (24.4)	596 (64.9)	99 (10.8)
Dementia	396 (43.1)	481 (52.3)	42 (4.6)
Geriatric assessment	128 (14.0)	555 (60.4)	236 (25.7)
Sarcopenia	160 (17.4)	593 (64.5)	166 (18.1)
Guidelines	140 (15.2)	670 (72.9)	109 (11.9)
Wishes of the family	360 (39.2)	537 (58.4)	22 (2.4)

We asked respondents to, "Please select the importance of each of the following assessment items when deciding surgical treatment methods for elderly cancer patients": (1) age; (2) PS; (3) judgment of anesthesiologist; (4) preoperative examination before treatment; (5) complications; (6) social background such as institutionalization or living alone; (7) presence of dementia; (8) overall evaluation of elderly patients; and (8) overall assessment of the elderly; (9) severity of sarcopenia; (10) guidelines; and (11) wishes of the family.

3: Very important; 2: Important; 1: Not important.

All respondents answered to these questionnaires. Most respondents answered that most items were "very important" or "important". In particular, > 50% of all respondents answered "very important" for PS, preoperative examination, and comorbidities. Social factors such as age, institutionalization and solitary residence, comprehensive evaluation of the elderly, severity of sarcopenia, and guidelines were "not important" for > 10% of all respondents. Of these, 25% and 18% chose "not important" for GA and sarcopenia, respectively.

**Figure 2:**
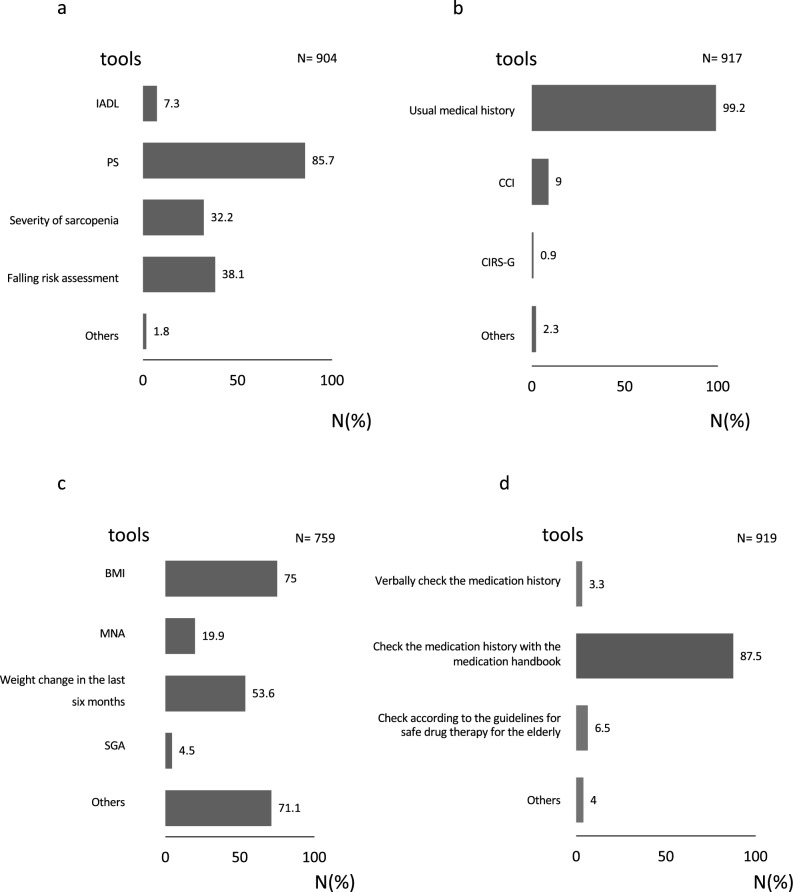

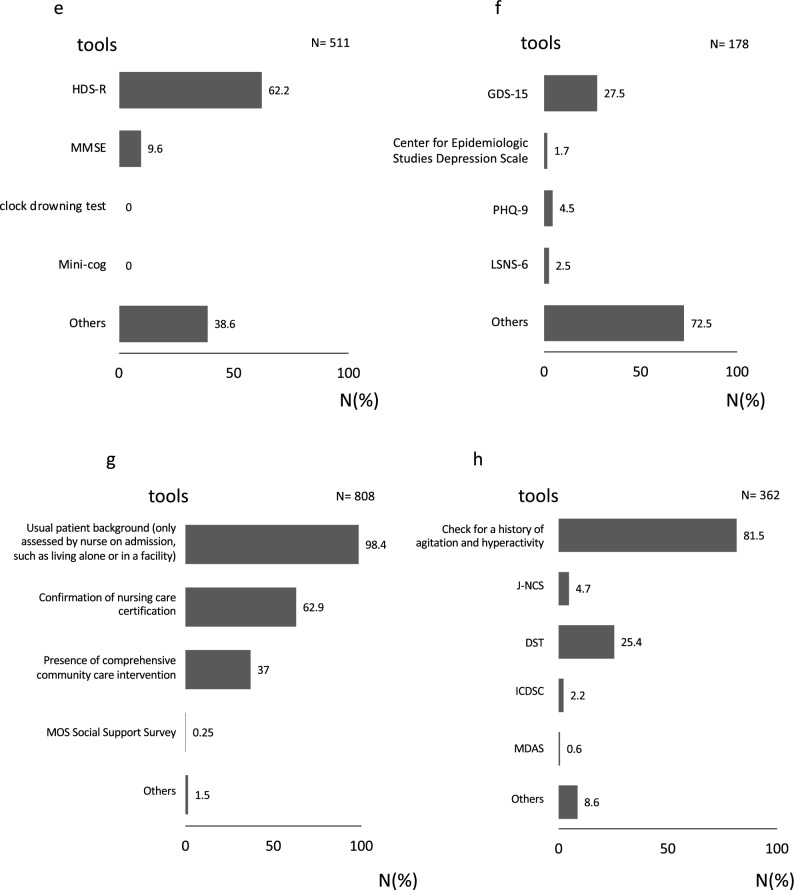
Evaluation tools for each preoperative evaluation item. We asked about the detailed evaluation methods for each preoperative evaluation item. (Multiple responses accepted). (**a**) Do you use specific tools to check the physical condition of patients before surgical treatment? (**b**) Do you use specific tools to check the complication status of patients before surgical treatment? (**c**) Do you use specific tools to check the nutritional status of patients before surgical treatment? (**d**) Do you use specific tools to check the medication status of patients before surgical treatment? (**e**) Do you use specific tools to check the cognitive status of patients before surgical treatment? (**f**) Do you use specific tools to check the mood of patients before surgical treatment? (**g**) Do you use specific tools to check the social support status of patients before surgical treatment? (**h**) Do you use specific tools to confirm the predicted disease onset before surgical treatment? *IADL* instrumental activities of daily living, *PS* performance status, *CCI* Charlson comorbidity index, *CIRS-G* cumulative illness rating scale-geriatric, *BMI* body mass index, *MNA* mini nutritional assessment, *SGA* subjective comprehensive assessment, *HDS-R* Hasegawa’s dementia scale-revised; *MMSE* mini-mental state examination; *GDS-15* geriatric depression scale-15; *PHQ-9* patient health questionnaire 9; *LSNS-6* Lubben social network scale-6; *MOS* medical outcomes study; *JNS* Japanese version of the NEECHAM confusion scale; *DST* delirium screening tool; *ICDSC* intensive care delirium screening checklist; *MDAS* memorial delirium assessment scale.

Preoperative assessment items and methods of evaluation (Table 4, Fig. 2).

**Table 4:** Questions regarding the implementation of each preoperative evaluation item.

	Assessed n (%)	Not assessed n (%)
Physical condition	904 (98.4)	15 (1.6)
Confirmation of complications	917 (99.8)	2 (0.2)
Nutritional condition	759 (82.6)	160 (17.4)
Cognition	511 (55.6)	408 (44.4)
Mood	178 (19.4)	741 (80.6)
Social support	808 (87.9)	111 (12.1)
Delirium	362 (39.4)	557 (60.6)

We asked respondents to indicate whether participants performed the following assessments before surgery: (1) physical condition; (2) confirmation of complications; (3) nutritional condition; (4) cognition; (5) mood; (6) social support; and (7) delirium. (Multiple responses accepted).

All respondents answered the questionnaire regarding implementation of the preoperative assessment items. Physical condition and comorbidity were assessed in almost all cases, followed by social support, nutritional status, and cognitive function in > 80% of cases. Delirium risk was assessed in 39% of cases, and mood state in 19% of cases (Table 4). The response rates of the additional questionnaires on the detailed evaluation methods were as follows: (a) physical condition: 98.4% (904/919), (b) comorbidities: 99.8% (917/919), (c) nutritional status: 82.6% (759/919), (d) medications: 100%, (e) cognitive status: 55.6% (511/919), (f) mood: 19.4% (178/919), (g) social support: 87.9% (808/919) and (h) risk of delirium: 39.4% (362/919). The specific methods of assessment most frequently used for each item were PS for physical function (86%), history taking for comorbidity (99%), body mass index (BMI) for nutritional status (75%), the revised Hasegawa Dementia Scale (HDS-R) for cognitive function (62%), usual patient background (assessment by nurses on admission) for social support status (98.4%) and confirming a history of agitation and hyperactivity for risk of delirium (81.5%) (Fig. 2).

## Discussion

This is the first large-scale survey to investigate the current status of preoperative assessment for elderly cancer surgery patients in Japan. Of these, survey cooperation was obtained from 919 oncological surgeons at 245 cancer treatment center hospitals nationwide. The survey revealed that not only is use of the GA not widespread, but awareness of GA also appears to be declining in medical education institutions. At the same time, the physical aspects of GA, such as assessment of physical functions and comorbidities, were widely conducted in each department with a common understanding, but assessment of nutritional status, cognitive functions, emotions/moods (depression, delirium), and social/economic status were not conducted sufficiently, and implementation varied among departments.

Elderly patients often have surgical risk factors such as physiological organ dysfunction, many comorbidities, poor nutritional status, impaired immunological function, and social limitations, which tend to increase the complications and severity of treatment. In a study by Simmonds et al. of 34,194 patients undergoing colorectal cancer surgery, mortality within 30 days after surgery increased with age, and was 6.2 times higher in patients over 85 years of age than in those under 65 years of age. Postoperative complications were more common and more severe in the elderly, including pneumonia, cardiovascular, cerebrovascular, and thrombosis^10^. Most importantly, since the elderly have really huge individual variances in reserve capacity, GA is intended to be used as a pre-treatment evaluation to identify problems unique to the elderly as well as chronological age and PS^3^. In recent years, the number of observational and case–control studies of GA in elderly cancer surgery patients has been increasing, and many reports have shown that GA as a preoperative assessment was useful in predicting postoperative complications^11–14^.

Despite these many informative reports, the most likely reasons for the low number of facilities performing GA as a preoperative screening test for elderly cancer patients in Japan and the low level of awareness of GA are as follows: (1) There are few high-quality randomized controlled trials that have verified the usefulness of GA in elderly cancer surgery patients, and guidelines have not been established. While several large observational studies have validated the predictive ability of GA, effective interventions to address the predicted risk remain unclear. Only one randomized controlled trial verifying the utility of preoperative GA has been reported. Ommundsen et al. randomized 122 frail elderly colorectal cancer patients to individualized interventions based on preoperative GA (e.g., treatment of comorbidities, nutrition therapy, medication reconciliation, delirium prevention, enhanced recovery after surgery) or usual care and compared the incidence of postoperative complications. The results showed that interventions based on preoperative GA did not reduce the incidence of grade II-IV adverse events^15^. Currently, there are several randomized controlled trials evaluating the usefulness of preoperative GA for elderly cancer surgery patients, and the results are promising^16,17^. However, differences in the background of the study population, selection bias, surgical techniques and assessment tools used, as well as endpoints, make it difficult to build evidence, and this is an issue that should be investigated in the future. (2) GA methods have been considered complicated and impractical. Since its evaluation requires special skills, it will take more than an hour to fully implement all domains of the GA. The SIOG proposes simplified screening methods such as Geriatric-8 and Vulnerable Elders Survey-13^6^. (3) The most important and fundamental problem is that there may be a lack of awareness of the importance of geriatrics among many oncologists in Japan. Nishijima et al. have recently clarified the current status of lack of geriatric medicine education in Japan^18^. In particular, due to the historical background of medical care being developed for emergency and specialty care, education in the field of geriatrics has lagged well behind, and medical care adapted to the elderly has often been thought to have been given insufficient consideration. Currently, only about 30% of universities in Japan have geriatric medicine departments, and education is in reality provided by a mixture of several fields^18^.

Additionally, this study showed that GA awareness is declining in medical education institutions. There are several possible factors that contributed to the differences in awareness and implementation of GA between medical education institutions and other hospitals. First, the implementation of GA to oncologic surgery in Japan has a strong research aspect. Some university hospitals in Japan are conducting clinical research of GA, and preoperative GA is routinely performed on a facility basis^19,20^. Secondly, as the background of the respondents, the response rate other than medical education institutions was about half that of medical education institutions (35.1% vs 65.6%). Thus, some of the department heads other than medical education institutions who participated in this survey may include a larger group of more interested in geriatrics, which may raise awareness of GA.

The strengths of this research are, firstly, that it is the first large-scale research in Japan targeting surgical departments. Secondary, there is little regional difference, and responses are obtained from the majority of facilities including the city centers and rural areas. Third, we were able to minimize data loss by refining the web response system. As a limitation, first, there was a large difference in the number of respondents between medical education institutions and other hospitals. In particular, respondents from hospitals other than medical institutions may include more doctors working on geriatric oncology, which may have caused a statistically significant difference in GA awareness. Secondly, the real respondents may not have been each department head. Although this study requested a survey of each department head at each institution, another doctor representing the department may responded without the intention of the department heads. There is a possibility that the number of years of experience as a doctor and the PhD acquisition rate do not accurately reflect the data of the department head. Third, GA implementation and postoperative outcome have not been examined. In order to clarify this, more detailed survey is required, which puts a heavy burden on the respondents. Since the purpose of this research was to investigate the current state of preoperative evaluation for elderly cancer patients, postoperative outcome was not set as a survey item. Despite some limitations, we believe that this study is a valuable resource that illustrates the current state of geriatric oncology surgery in Japan. We believe that this study provides fundamental data to validate the establishment of an appropriate preoperative evaluation method.

In conclusion, this large questionnaire survey on the current status and problems of elderly cancer surgery patients in Japan revealed not only that GA is not widespread, but also that the level of awareness of GA in medical education institutions is low. The practice of geriatrics and its education seem to be in question. To address this serious situation, accumulation of evidence on the treatment of elderly cancer patients in Japan and establishment of treatment guidelines are urgent tasks. Based on the present results, the relationship between GA and postoperative prognosis for elderly cancer surgery patients needs to be verified in Japan to establish appropriate methods of pretreatment evaluation for elderly cancer patients.
