# Author Correction: A nationwide web-based survey of oncologic surgeons to clarify the current status of preoperative assessment for elderly cancer surgery patients in Japan

**DOI:** 10.1038/s41598-023-36673-5

**Published:** 2023-06-15

**Authors:** Daisuke Inoue, Makoto Yamamoto, Hisatomi Arima, Kazuo Tamura, Yoshio Yoshida

**Affiliations:** 1 Department of Obstetrics and Gynecology, University of Fukui, 23-3 Matsuoka-Shimoaizuki, Eiheiji-Cho, Yoshida-Gun, Fukui 910-1193 Japan; 2 Department of Preventive Medicine and Public Health, Fukuoka University, 7-45-1 Nanakuma, Jhonan-Ku, Fukuoka, Fukuoka Japan; 3 Emeritus Professor, Faculty of Medicine, Fukuoka University, 7-45-1 Nanakuma, Jhonan-Ku, Fukuoka, Fukuoka Japan

Correction to: *Scientific Reports* 10.1038/s41598-021-02319-7, published online 23 November 2021

The original version of this Article contained an error in the Results section, where

“We asked each department of the 436 facilities to cooperate in completing the questionnaire, and received responses from 245 facilities (56%), with a total of 941 department heads. Of these, 32 department heads declined to participate in the study and valid responses were obtained from 919 department heads.”

now reads:

“We asked each department of the 436 facilities to cooperate in completing the questionnaire, and received responses from 245 facilities (56%), with a total of 941 department heads. Of these, 22 department heads declined to participate in the study and valid responses were obtained from 919 department heads.”

The original Article has been corrected.

